# Early versus standard return to play following ACL reconstruction: impact on volume of play and career longevity in 180 professional European soccer players: a retrospective cohort study

**DOI:** 10.1186/s10195-025-00837-y

**Published:** 2025-05-12

**Authors:** Michael Battaglia, Justin W. Arner, Kaare S. Midtgaard, Daniel B. Haber, Liam A. Peebles, Annalise M. Peebles, Phob Ganokroj, Ryan J. Whalen, Matthew T. Provencher, Guglielmo Torre, Riccardo Ciatti, Pier Paolo Mariani

**Affiliations:** 1 Villa Stuart Sport Clinic-FIFA Medical Centre of Excellence, Rome, Italy; 2 Department of Orthopaedic Surgery, University of Pittsburgh Medical Center, Pittsburgh, PA USA; 3 Division of Orthopaedic Surgery, Oslo University Hospital, Oslo, Norway; 4 Panorama Orthopedics and Spine Center, Denver, CO USA; 5 Steadman Philippon Research Institute, Vail, CO USA; 6 Faculty of Medicine Siriraj Hospital, Mahidol University, Bangkok, Thailand; 7 The Steadman Clinic, Vail, CO USA; 8 Department of Movement, Human and Health Sciences, University of Rome Foro Italico, Rome, Italy; 9 Università San Raffaele, Rome, Italy

**Keywords:** Knee, Ligaments, ACLR, Football (soccer), Physical therapy/rehabilitation, Return-to-play

## Abstract

**Background:**

Patients typically follow a 7–9-month return to play (RTP) protocol following anterior cruciate ligament reconstruction (ACLR); however, much of these data have been based on non-elite athletes. The purpose of this study is to understand whether professional soccer players returning to competition < 6-months following ACLR will have an increased risk of graft failure, play fewer seasons postoperatively, and have lower volume of play compared with those returning > 6 months.

**Materials and methods:**

A total of 180 male professional European soccer players were enrolled and underwent ACLR with a single surgeon between April 2008 and December 2016 and returned to sport < 6 months (early RTP group, *n* = 92) or > 6 months (standard RTP group, *n* = 88). Time from intervention to RTP (days), same season returns, total games and average minutes played in return season, seasons played after surgery, and playing status were recorded.

**Results:**

The early RTP group returned to soccer sooner (142.8 ± 21.4 days) than the standard RTP group (276.2 ± 118.9) (*p* < 0.01), and more players returned the same season as the injury in the early RTP group (*n* = 55/92, 62.5%) than the standard RTP group (*n* = 18/88, 20.5%) (*p* < 0.01). The difference in average minutes per game in the first season back was not statistically significant (early RTP, 56.7 ± 22.3 min; standard RTP 49.9 ± 29.8 min, *p* = 0.094). The early RTP group had significantly longer careers following ACLR (5.7 ± 2.2 seasons) than the standard RTP group (4.7 ± 2.4 seasons) (*p* = 0.005). The early RTP group sustained more reruptures (*n* = 4, 4.4%) than the standard RTP group (*n* = 1, 1.1%).

**Conclusions:**

Professional European soccer players returning to competition < 6 months following ACLR did not have poorer outcomes than those who returned > 6 months despite the fact that there were three more failures. However, the early RTP group players were more likely to return during the same season, had longer careers after ACLR, and played a similar number of games and minutes per game, but had more graft failures.

**Level of evidence:**

Retrospective cohort study level IV.

*Trial registration*: Retrospectively registered according to prot. Professionisti_OSS_22.

**Graphical Abstract:**

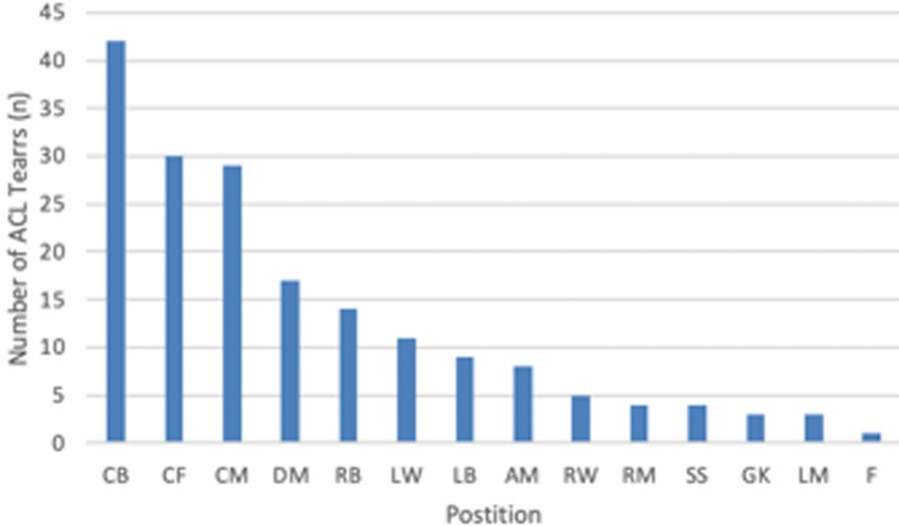

**Supplementary Information:**

The online version contains supplementary material available at 10.1186/s10195-025-00837-y.

## Introduction

There is a paucity of scientific literature regarding the ideal return to professional sports following anterior cruciate ligament reconstruction (ACLR). It is known that a higher activity level leads to a greater risk of ACL graft failure, but many other factors are also at play [13]. Basic science studies suggest that ACL graft maturation and re-ligamentization are critical; however, these processes continue to occur after athletes actually return to play, with some studies reporting changes up to 24 months [19, 23]. The time for the optimal return to play is, therefore, controversial, with most advocating for between 6 and 12 months. Recently published data have suggested that lower failure rates may be obtained 9 months after ACLR [10]. Despite this, several factors must be considered regarding return to play (RTP) in addition to timing. These include range of motion, proprioception, strength, and isokinetic and functional testing, as well as psychological readiness [20, 22].

Although an inadequate RTP protocol carries an increased risk of reinjury, many professional athletes desire to push the envelope to avoid loss of position, decline in conditioning and skill level, and loss of income. However, professional athletes likely have greater neuromuscular control, proprioception, and strength compared with non-elite athletes. Further, these athletes typically have greater access to high-level and more frequent physical therapy, recovery resources, technology, and nutrition, leaving little applicability to many of the currently published studies [18]. Further, elite athletes are typically more likely to continue important injury prevention exercises throughout their play [5, 22]. Conversely, professional sports, including soccer, are likely riskier due to high levels of jumping, pivoting, and hard cutting [10]. This begs the question of whether professional athletes should be restricted from returning to play for 9 months, as many surgeons currently recommend, which is based on data from non-elite athletes.

## Materials and methods

### Aim of the study, design, and setting

The aim of this study was to retrospectively evaluate the impact of early (< 6 months) versus standard (> 6 months) return to play (RTP) protocols on the rate of failure, volume of play, and career longevity following ACLR in a cohort of professional European soccer players. It was hypothesized that athletes returning to professional competition earlier than 6 months after ACLR would have an increased risk of failure, lower volume of play, and would play for fewer seasons following surgical intervention compared with those returning after 6 months.

Professional soccer players treated with primary anterior cruciate ligament reconstruction by a single surgeon (P.P.M.) between April 2008 and December 2016 were included in this retrospective study. The study was approved by the institutional review board (prot. Professionisti_OSS_22), and all study patients signed a consent form following written and oral information for the management of clinical data for research purposes. Eligible patients were treated at the study center from April 2008 to December 2016, with continued data regarding the patients’ ACLR failure and professional soccer status recorded through April 2023.

### Study population

The inclusion criteria were that the patient was (1) male and a (2) professional soccer player undergoing a (3) primary anterior cruciate ligament reconstruction. The exclusion criteria were (1) ipsilateral prior ACL or ligamentous knee injury, (2) previous injury requiring surgery on the same limb, including fractures. The cohort was then divided into two groups on the basis of the time to RTP, defined as the time of the first match participation after surgery: the “Early RTP” group for those players returning to a match before 180 days from surgery and “Standard RTP” group for those returning after 180 days. The postoperative physical therapy accelerated protocol was the same for all the patients.

### Data extraction and management

The patients’ position was recorded and categorized into (1) goalkeeper, (2) defensive player (e.g., center back, full back), (3) midfielder, and (4) attacker (e.g., forward, winger). The player’s club and league level at the time of injury were recorded.

### Surgical technique

All patients were treated surgically with arthroscopic evaluation prior to autograft harvest. No allograft was used as it is not readily and safely available in Europe. The preference was for ipsilateral bone-patellar tendon-bone autograft. A notchplasty was performed in all cases to allow for proper positioning of the femoral tunnel through a tibial tunnel at the 10 o’clock position for right knee or 2 o’clock for left knee. Suspensory fixation was utilized for the femoral side and an interference screw (bioabsorbable) for the tibial side. Meniscal pathology, including root tears, were treated preferentially with a repair. 

### Rehabilitation protocol

All patients were admitted to the hospital overnight and began physical therapy postoperatively on day one. Patients were followed at the physiotherapy center for a minimum of 4 weeks after surgery prior to returning to their team physician, who supervised therapy and ultimately determined when the patients were permitted to return to play. For the first 4 weeks, the subjects worn a knee brace locked in full extension for the first 2 weeks and then locked 0–90°. Full weight bearing was allowed, unless a medial meniscal suture at the posterior horn (not including ramp lesions) or suture of bucket handle tears of the lateral meniscus. Exercises consisted in progressive resistance isometric strengthening for quadricep and flexor muscles, bike with progressive resistance, and stretching, with the progressive recovery of full ROM. Once the players returned to their team physician, rehabilitation protocol was not standardized but was in charge to the team physiotherapy staff. The treating surgeon was not involved in the rehabilitation program once the patient returned to their respective team physician, nor was the treating surgeon involved in the decision for timing of return to play. However, some key requirements must be met before the athlete was cleared for field rehabilitation by the surgeon: 20% limb symmetry index (LSI) for isometric maximal voluntary contraction of the quadricep, 10% LSI for countermovement jump peak ground reaction force, and 10% LSI for triple hop test. Ultimately, the respective team physician was responsible for the rehabilitation and return to play criteria for each patient.

Time from intervention to return to the first official game was recorded in days. Information if the patient returned in the same season was recorded, as well as the number of games and total and average minutes played in the return season. The number of seasons played after injury and if the player was active at the time of data collection (September 2019) were also recorded. Failure of ACLR, defined as a reinjury to the reconstructed ACL that required revision surgery, was also recorded.

### Statistical analysis

Normality of continuous data was tested by visual inspection of histograms, QQ-plots, and the Shapiro–Wilk test. Continuous data were described with mean and standard deviation (SD), and median, range, frequency, and percentage were used for categorical data. Differences between groups were analyzed with the chi-squared test for categorical data and with Student’s *t*-test or the Mann–Whitney *U* test for continuous data. The statistical software, R, version 4.0.2, was used for all plots and analyses (access date 22 June 2020; R Core Team, Vienna, Austria, with the additional package ggplot2). The level of significant difference was set at 0.05.

## Results

**Fig. 1:**
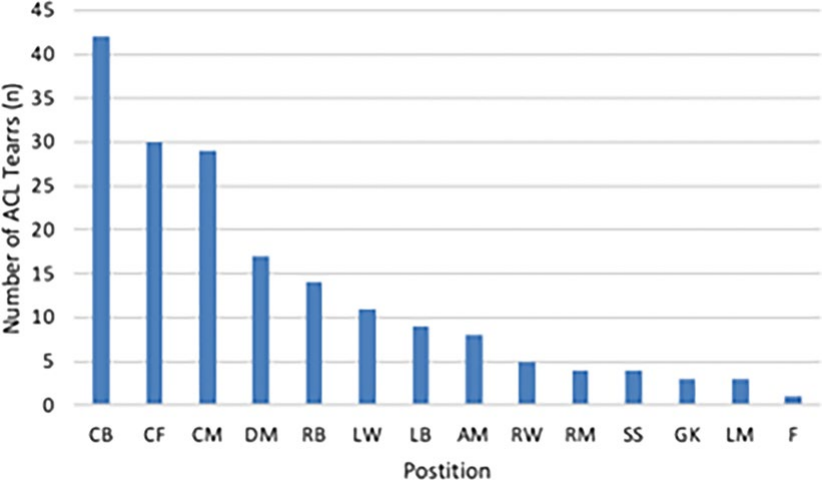
Patient demographics by soccer position. *CB*  center back, *CF*  center forward, *CM*  center midfielder, *DM*  defensive midfielder, *LW*  left wing, *LB*  left back, *AM* attacking midfield, *RW*  right wing, *RM*  right midfielder, *SS*  second striker, *GK*  goalkeeper, *LM*  left midfielder, *F*  forward

A total of 180 players were included in this study (mean age, 24.2 years old; range, 17–36 years old, Table 1). The distribution of player positions is shown in Fig. 1. A total of 92 early RTP (51.1%) and 88 standard RTP players (48.9%) were identified. Players in the early RTP group returned to professional-level soccer at a mean of 142.8 ± 21.4 days, which was significantly sooner than players in the standard RTP group who returned at a mean of 276.2 ± 118.9 days (*p* < 0.01) following ACLR (Table 2). A significantly greater number of players in the early RTP group returned during the same season of intervention (*n* = 55/92, 62.5%) than in the standard RTP group (*n* = 18/88, 20.5%) (*p* < 0.01). Players in the early RTP group competed in an average of 11.5 ± 9.5 games during their return season, while those in the standard RTP group competed in an average of 13.0 ± 11.0 games (*p* = 0.736). The early RTP group played, on average, a total of 763.9 ± 806.3.2 min and 56.7 ± 22.3 min/game in their return season, while the standard RTP group played an average of 812.8 ± 818.12 total minutes and 49.9 ± 29.8 min/game in their return season (*p* = 0.98 and *p* = 0.09, respectively). Players in the early RTP group had significantly longer careers following ACLR, averaging 5.7 ± 2.2 seasons, compared with those in the standard RTP group, who competed in an average of 4.7 ± 2.4 seasons after surgical intervention (*p* = 0.005) (Table 2). The early RTP group had four (4.4%) failures, while the standard RTP group had one (1.1%) failure.

**Table 1:** Demographic data

Parameters	Early RTP (*n* = 92); *n* (%)	Standard RTP (*n* = 88); *n* (%)	*p*-Value
Age at intervention (years)	24.7 ± 4.0 25.0 (22.0–27.0)	23.9 ± 4.7 23.0 (20.0–27.0)	0.177
Interventions			
Medial meniscus resection	5 (5.7%)	7 (8%)	0.766
Medial meniscal repair	20 (22.7%)	25 (28.4%)	0.490
Lateral meniscus resection	25 (28.4%)	23 (26.1%)	0.866
Lateral meniscus repair	25 (28.4%)	28 (31.8%)	0.743
Articular cartilage lesion	11 (12.5%)	12 (13.6%)	> 0.99
Additional intervention	12 (13.6%)	15 (17%)	0.676
Medical complications	2 (2.3%)	6 (6.8%)	0.278

**Table 2:** Comparison of the return to play parameters following ACL reconstruction for professional European soccer players between early and standard RTP group

Parameters	Early RTP (*n* = 92); *n* (%)	Standard RTP (*n* = 88); *n* (%)	*p*-Value
Return to soccer (days)	142.8 ± 21.4 143.0 (127.0–159.0)	276.2 ± 118.9 242.5 (198.5–301.0)	< 0.01*
Return during the same season as intervention?	55 (62.5%)	18 (20.5%)	< 0.01*
Number of games played in return season	11.5 ± 9.5 8.0 (4–17)	13.0 ± 11.0 10.0 (3–21)	0.736
Total minutes in return season	763.9 ± 806.3 552.5 (184–1003)	812.8 ± 818.12 615.0 (73–1363)	0.981
Average minutes per game in return season	56.7 ± 22.3 55.7 (44.4–78.2)	49.9 ± 29.8 55.0 (28.1–73.7)	0.094
Post-return seasons played after intervention	5.7 ± 2.2 5.0 (4–7)	4.7 ± 2.4 5.0 (3–6)	0.005*

*RTP*  Return to play

*Statistically significant

Additional intervention, other concomitant surgical procedures to ACL reconstruction such as collateral ligament repair or augmentation. Early return to play (RTP), less than 180 days, and standard RTP, more than 180 days. Age at intervention is represented by mean ± standard deviation and median (interquartile range).

## Discussion

The most important finding of this study is that players in the early RTP group returned to soccer significantly sooner, a greater number returned during the same season of intervention, and had longer careers (number of seasons) following ACLR compared with the standard RTP group. Despite these findings, there was a higher number of graft failures in the early RTP group. A previous systemic review from Ardern et al. demonstrated that 80% of people returned to any sport, and only 55% returned to the competitive level after ACLR for non-elite athletes [1]. However, professional and elite athletes will have differences in physical fitness and psychological and social aspects compared with the general population [15]. The recent systemic review and meta-analysis from Lai et al. showed a higher RTP rate of 85% in elite soccer players following ACLR [15]. From their study, the time taken to RTP varied depending on surgeon preferences and type of sports; on average, soccer players returned to sport between 6 and 10.2 months postoperatively [6, 12, 15, 21, 24]. A case–control study from Longstaffe et al. studied the RTP and career length after ACLR among professional Canadian soccer players and found an overall of 69% were able to RTP at least one game with a mean RTP of 10.4 months. [16] For those who could RTP, the mean career length was 2.8 seasons (34.4 games). Another Cohort study from Forsythe et al. also demonstrated a high RTP rate (80%) of elite Union of European Football Associations (UEFA) soccer leagues and an RTP time of 216 days (7.2 months) [8]. They also reported that the injured athletes had significantly inferior performance (fewer games and minutes per season) for the first two seasons after injury (*p* < 0.001); however, the players’ performance was equaled or exceeded that of their matched controlled after season three [8]. The finding from Barth et al. also supported that the player performance did not return to their baseline number of games per season and minutes per game until three seasons following ACLR [2]. Compared with the findings in our study, 60.9% of the early RTP group returned during the same season of intervention. Players in our cohorts had longer careers, with 5.7 and 4.7 seasons following ACLR for the early and standard RTP groups, respectively. The type and level of sports participation influence the time for athletes to return to the preinjury level [17]. Time taken for the professional American football players (on average 8.2–13 months) and basketball players (10.7–11.8 months) to return to the preinjury level were longer compared with the soccer players (6–10.2 months) [15]. While the mean RTP time in rugby is shorter (within 6 months after surgery), there was a small number of cases, and only half of this cohort had normal functional outcome (IKDC) at 2 years follow-up period [7, 15]. 

Gender, age, side of injury, or graft choice may be related to the rate of return to play and reinjury of the ACL. The injury risks following ACLR in soccer athletes from the prospective Multicenter Orthopaedic Outcomes Network (MOON) group demonstrated that female athletes were more likely to have a future ACL surgery (20% versus 5.5%, *p* = 0.03), and surgery in non-dominant limb had a higher rate of contralateral ACLR than previous surgery at the dominant limb (16% versus 3.5%, *p* = 0.03) [3]. Their results also demonstrated that older athletes and female athletes were less likely to return to sport [3]. A large cohort study from King et al. showed an overall high RTP rate of 81% at a mean F/U period of 28.4 months, and those who returned had a retear of 2.7% of all ACLRs, which was lower for the bone-patellar tendon graft (BPTB) of 1.3% compared with hamstring graft (HT) of 8.3% [14]. There was a superior survival rate of the BPTB graft relative to the HT graft after surgery (*p* < 0.001) [14]. Therefore, BPTB demonstrated a lower reinjury rate in level I athletes. Our study also demonstrated a graft failure rate of 2.8%, which was similar to the cohorts from King et al., and there was a higher number of failures in the early RTP group compared with the standard RTP group.

An effective rehabilitation is a key component for a successful ACLR. An accelerated rehabilitation protocol has been introduced with the goal of achieving early full range of motion and commencing strengthening exercises at the same time. Gupta et al. conducted a prospective randomized controlled trial (RCT) that compared accelerated rehabilitation and standard rehabilitation after ACLR using HT autograft. They demonstrated equivalent in terms of laxity, patient satisfaction, functional performance, and activity level and better in terms of clinical outcome (IKDC scores at 3 and 6 months; and KOOS scores at 3 months, *p* < 0.05) to a standard rehabilitation protocol [11]. A recent systemic review of ACL recovery and rehabilitation by Glatke et al. demonstrated that accelerated rehabilitation can be effective for patients who underwent ACLR using HT autograft [9]. Our study also chose the same accelerated rehabilitation protocol for both groups, starting on day 1 after surgery. Patients were encouraged to participate in therapy for at least 4 weeks before returning to their team physician. Time to RTP is one of the considerable risk factors for reinjury of the ACL. A recent meta-analysis of risk factors for graft rupture following ACLR demonstrated no association between RTP time and graft rupture [4]. Likewise, a large prospective study from King et al. also demonstrated no relationship between time to RTP and secondary ACL injury [14].

The current study had several limitations. Firstly, the authors recruited all-male professional soccer players for this study. Therefore, the findings of this study cannot be fully generalized to the general population, especially female athletes. Secondly, the authors preferred BPTB autograft for graft selection in this study. A future study to focus on differences between early RTP and standard RTP after ACLR using other graft choices, such as hamstring or quadriceps tendon autograft or allograft, should be conducted for clinical application. Finally, several factors other than timing, such as range of motion, muscle strength, proprioception, or psychological readiness, were not determined in this study regarding RTP. Further analysis of these factors associated with early RTP or standard RTP would be helpful.

## Conclusions

Professional European soccer players returning to competition < 6 months following ACLR did not appear to have poorer outcomes than those who returned > 6 months, but there were 3 (3.3%) more failures. However, the early RTP group patients were more likely to return to play during the same season as their injury, had longer careers following the surgery and played a similar number of games and minutes per game in their return season. These advantages and disadvantages of the early RTP protocol should be discussed with the athletes individually, the agents, and the team coaches for the benefit of each individual player.

## Supplementary Information

Supplementary Material 1.

## Data Availability

No additional data are available. Data are available on reasonable request to the corresponding author.

## Abbreviations

ACL: Anterior cruciate ligament
ACLR: Anterior cruciate ligament reconstruction
RTP: Return to play
