# Supplementary material for: Early versus standard return to play following ACL reconstruction: impact on volume of play and career longevity in 180 professional European soccer players: a retrospective cohort study
Source: J Orthop Traumatol. 2025 May 12;26:29. doi: 10.1186/s10195-025-00837-y (PMC12069203; doi:10.1186/s10195-025-00837-y)
Supplement: Supplementary file 1 — Supplementary Material 1. [file 10195_2025_837_MOESM1_ESM.pdf]

DECLARATIONS

Ethics approval and consent to participate: the study was approved by the institutional review board (prot. Professionisti_OSS_22)

Consent for publication: not applicable

Availability of data and material: no additional data are available. Data are available on reasonable request to the corresponding author.

Competing interests: the authors declare that they have no competing interests

Funding: not applicable

Authors' contribution:

Michael Battaglia: conceived and designed the analysis
Justin W. Arner: wrote the paper
Kaare S. Midtgaard: stats analysis
Daniel B. Haber: wrote the paper
Liam A. Peebles: wrote the paper
Annalise M. Peebles: critical revisions
Phob Ganokroj: critical revisions
Ryan J. Whalen: critical revisions
Matthew T. Provencher: conceived and designed the analysis
Guglielmo Torre: critical revisions
Riccardo Ciatti: critical revisions and submission according to guidelines of the journal
Pier Paolo Mariani: conceived and designed the analysis

Acknowledgements: not applicable
